# The Current Consideration, Approach, and Management in Postcesarean Delivery Pain Control: A Narrative Review

**DOI:** 10.1155/2021/2156918

**Published:** 2021-09-18

**Authors:** L. Sangkum, T. Thamjamrassri, V. Arnuntasupakul, T. Chalacheewa

**Affiliations:** Department of Anesthesiology, Faculty of Medicine, Ramathibodi Hospital, Mahidol University, Bangkok 10400, Thailand

## Abstract

Optimal postoperative analgesia has a significant impact on patient recovery and outcomes after cesarean delivery. Multimodal analgesia is the core principle for cesarean delivery and pain management. For a standard analgesic regimen, the use of long-acting neuraxial opioids (e.g., morphine) and adjunct drugs, such as scheduled acetaminophen and nonsteroidal anti-inflammatory drugs, is recommended unless contraindicated. Oral or intravenous opioids should be reserved for breakthrough pain. In addition to the aforementioned use of multimodal analgesia, preoperative evaluation is critical to individualize the analgesic regimen according to the patient requirements. Risk factors for severe postoperative pain or analgesia-related adverse effects will require modifications to the standard analgesic regimen (e.g., the use of ketamine, gabapentinoids, or regional anesthetic techniques). Further investigation is required to determine analgesic drugs or dose alterations based on preoperative predictions for patients at risk of severe pain. Outcomes beyond pain and analgesic use, such as functional recovery, should be determined to evaluate analgesic treatment protocols.

## 1. Introduction

The rate of cesarean delivery has been increasing over the past decades, and it is one of the most commonly performed surgeries in the world, with nearly 18.5 million cesarean deliveries performed annually [1]. The causes to explain this higher trend including an increase in cesarean performed for maternal request, increased number of high-risk expectant mothers, changes in provider practice patterns, and the obstetrical medicolegal environment [2, 3].

Pain following cesarean delivery is a complex experience that is personalized to each patient. The degree of tissue injury triggers a response in the pain matrix, forming peripheral sensitization and central pain pathways to fear, anxiety, and frustration. Patients have reported concerns about pain during and after cesarean delivery as their highest priority [4]. The intensity and duration of pain experience increase the likelihood of greater opioid use, delayed recovery [5], and impeded maternal and fetal bonding [6]. Furthermore, severe acute pain is a strong risk factor for postpartum depression and chronic pain [7, 8], which results in long-term psychological, social, and economic adversities [9, 10]. Therefore, optimal pain control is a key priority on both humanitarian grounds and for efficient health service delivery [11–13].

In addition to improving clinical outcomes and functional recovery. Enhanced recovery after surgery has been shown to lead to a reduction in complications and duration of hospital stay, as well as earlier resumption of normal activities [14]. Optimal pain control is a cornerstone of enhanced recovery after cesarean delivery (ERAC) [15, 16], and it is an essential component of the Obstetric Quality-of-Recovery (ObsQoR-10) score [17–19].

To optimize pain control with faster recovery and fewer side effects, stepwise multimodal analgesia is crucial for the management of postoperative pain. However, perioperative pain management should be individualized according to patient conditions (e.g., a history of chronic pain) or anesthetic techniques (general anesthesia or neuraxial anesthesia). This narrative review presents key considerations and approaches to the management of postoperative pain in cesarean delivery.

## 2. Identification of Women at Risk of Severe Postoperative Pain

To optimize postoperative analgesia, pain management protocols have moved toward a standardized approach to personalized analgesic management. A large cohort study assessed pain resolution, opioid-free status, and functional recovery after vaginal and cesarean delivery. The time to pain resolution after delivery varied between 0 and 85 days [5]. This finding suggests that a standardized approach is not appropriate for the entire postpartum population and that pain management should target women at risk of severe or prolonged pain.

Several studies have evaluated patient risk factors during the preoperative period, including demographic and psychological factors and quantitative sensory tests (QSTs). In patients undergoing general surgery, female sex, younger age, preoperative anxiety, and a history of chronic pain were significant predictors of worse postoperative pain [20]. In patients undergoing cesarean delivery, several studies have investigated the role of preoperative QSTs or pain response to local anesthetic infiltration in predicting acute postoperative pain [21]. The correlations of preoperative QSTs (pressure, thermal, and electrical) with postoperative pain outcomes were weak to modest in most studies [22, 23]. Therefore, the clinical role of preoperative QSTs is limited. The pain score upon local anesthetic infiltration was modestly associated with acute postoperative pain [21] as well as subacute postoperative pain [24]. Three simple questionnaires assessing anxiety, anticipated pain, and analgesic requirements were used to predict the upper 20^th^ percentile of the evoked pain score. The results revealed modest sensitivity (68%) and specificity (67%) [25]. However, the clinical use of the three simple questionnaires combined with the pain response to local anesthetic infiltration is easy to apply and may provide some value.

Another approach is giving patients more of a role in analgesic regimen selection. In a randomized controlled trial study, patients were selected to receive either high-dose (200 mcg) or low-dose (100 mcg) intrathecal morphine based on information regarding pain relief and side effects [26]. The results revealed that patients who requested the larger dose required more supplemental opioids and reported more pain than those who requested the smaller dose. Another study reported similar results, with patients choosing a higher dose (300 mcg intrathecal morphine + single dose oral gabapentin 600 mg) requiring more rescue opioids than those selecting a medium dose (150 mcg) or low dose (50 mcg) [27]. This finding confirmed that patients had insight into their pain needs. Patient-centered analgesic management may provide better patient expectations and outcomes based on individual preferences for pain relief and avoidance of side effects. Risk factors for severe postoperative pain after cesarean delivery are given in Table 1.

## 3. Special Concerns about Pain Control in Cesarean Delivery

Compared with other procedures, optimal pain control in cesarean delivery involves several key considerations:

1. Preemptive analgesia is limited by concerns in utero fetal drug transfer
2. The anesthetic technique is exclusively neuraxial anesthesia
3. Potential analgesic drug transfer to breastfeeding neonates should be considered. Opioids are associated with breast milk transfer and may cause neonatal sedation. Therefore, opioid-sparing multimodal analgesia is preferable.
4. The transition to oral medications as soon as possible is preferred. Early mobilization and enhancing the mother's ability to be independent and to care for her newborn baby is critical.

To achieve effective analgesia, postoperative opioid requirements and side effects should be decreased. Postcesarean delivery analgesia may be enhanced by many intraoperative interventions for multimodal analgesia, such as neuraxial opioids, nonopioid analgesics, regional blocks, or local analgesia infiltration.

### 3.1. Neuraxial Opioids

Neuraxial anesthesia is the preferred anesthetic technique for cesarean delivery [34]. Neuraxial anesthesia decreases maternal risk and improves fetal outcomes with the additional benefit of superior postoperative analgesia with the use of neuraxial opioids [35].

Neuraxial morphine binds to G-protein-like pre and postsynaptic opioid receptors in the dorsal horn, causing potassium channel opening and calcium channel closure, with an overall reduction in intracellular calcium. This reduces glutamate and substance P release from presynaptic C fibers and decreases nociceptive transmission [36]. In addition, neuraxial morphine spreads cephalad and binds to opioid receptors in the brain stem that indirectly activate the descending pain pathway, thus mitigating pain signaling [37].

### 3.2. Intrathecal Morphine

Intrathecal morphine is the gold standard single-shot drug for postcesarean pain. The duration of action of intrathecal morphine is between 14 and 36 h [38]. A meta-analysis revealed that high-dose intrathecal morphine (100–250 mcg) prolonged analgesia after cesarean delivery compared with low-dose intrathecal morphine (50–100 mcg) by 4.5 h (95% confidence interval (CI), 1.9–7.1). Both groups had comparable pain scores and 24 h morphine consumption. However, a lower dose of intrathecal morphine was associated with a lower incidence of nausea or vomiting (OR, 0.44; 95% CI, 0.27–0.73) and pruritus (OR, 0.34; 95% CI, 0.2–0.59) [39]. None of the studies in this meta-analysis reported respiratory depression in any of the patients.

As part of multimodal analgesia, a randomized double-blinded control study determined the dose response of intrathecal morphine when administered with intravenous ketorolac. The results suggested that 50 mcg intrathecal morphine produces analgesia similar to that produced by either 100 mcg or 150 mcg [40]. In summary, increasing doses of intrathecal morphine extended the analgesic duration following cesarean delivery but increased the risk of side effects (e.g., nausea and itching). Using intrathecal morphine as part of a multimodal analgesic regimen, the optimal dose of intrathecal morphine is between 50 and 100 mcg.

### 3.3. Epidural Morphine

Eventhough most cesarean deliveries are performed mainly with spinal anesthesia [41], unplanned cesarean deliveries are often performed on patients in labor with epidurals in situ. For these patients, epidural catheters can be used for the administration of epidural morphine. However, the optimal dose of epidural morphine is unclear, and dosing has been based on intrathecal morphine equivalency studies and dose-finding studies. Equipotent dosing (equianalgesic dose) requires a conversion ratio of 20 : 1–30 : 1 between epidural and intrathecal administration [42, 43]. The optimal dose was 3 mg in a large retrospective study [44] and 3.75 mg in a dose-response study [45]. In a randomized controlled trial study of 87 elective cesarean deliveries under combined spinal epidural anesthesia, 24 h opioid consumption of epidural morphine 1.5 mg and 3 mg was compared. No significant difference was observed in postcesarean delivery analgesia between the groups, but epidural morphine 1.5 mg led to fewer side effects. However, this study included acetaminophen and ketorolac as part of the multimodal regimen, which may have mitigated the analgesic differences between the lower and higher epidural morphine dose groups [46].

Neuraxial morphine is well known for its high-quality postcesarean delivery, pain control, simplicity of administration, and cost-effectiveness [38]. Therefore, neuraxial morphine is currently regarded as the gold standard for analgesia following cesarean delivery. However, neuraxial morphine has known side effects. To minimize dose-related adverse effects, the optimal dose is a balance between optimal analgesia and minimal side effects. The ideal dose for a “single-shot” intrathecal dose appears to be 50–100 mcg, and the “single-shot” epidural morphine dose is 1.5–3 mg when used in multimodal analgesia.

### 3.4. Regional Blocks for Cesarean Section

Regional anesthesia is strongly advocated within a nonobstetric surgical setting as part of multimodal analgesic strategies [47]. As part of the ERAC program, if neuraxial morphine cannot be administered, regional anesthesia plays a significant role in postoperative analgesia. Regional anesthesia improves analgesia and decreases postoperative opioid requirements. Moreover, the use of regional anesthesia may be beneficial to provide relief from severe incisional pain or for patients at risk for severe acute pain [48]. There are several regional anesthesia techniques as follows.

### 3.5. Local Anesthetic Wound Infiltration and Infusion

Local anesthetic wound infiltration and infusion are alternative strategies to reduce IV and oral opioid consumption and decrease opioid-related side effects. A meta-analysis included single-shot and continuous wound infusion in patients undergoing cesarean delivery with and without intrathecal morphine. The results showed that these techniques provided an opioid-sparing effect (mean difference −9.69 mg of morphine equivalents (95% CI −14.85 to −4.52)) but had a minimal effect on pain scores (mean difference −0.36, 95% CI −0.58 to –0.14) [49]. In the subgroup analysis, 24 h morphine consumption and 24 h pain score at rest and with movement were significantly decreased in patients who did not receive intrathecal morphine. However, 24 h pain scores with movement and 24 h morphine consumption had no statistically difference in patients who received intrathecal morphine. Therefore, the addition of anesthetic wound infiltration and infusion in patients who received intrathecal morphine seems to have limited benefit [50].

Single-shot wound infiltration in cesarean delivery has a limited analgesic duration of 4–12 h [51, 52]. Therefore, continuous wound infiltration is preferred over a single injection. With respect to the catheter placement site, subfascial catheters are preferred over above-fascial catheters, as they showed lower pain at rest and less total morphine consumption than above-fascial catheters [53]. The hypothesized better outcomes in the use of subfascial catheters are probably due to an anti-inflammatory effect of the local anesthetic, which is absorbed intraperitoneally, and less leakage with subfascial infusion. Various agents and infusion parameters have been studied in cesarean delivery, such as continuous infusion vs. intermittent infusion or the addition of NSAIDs to the local anesthetic [50, 54–56]. To date, the optimal agents, dose of local anesthesia, and infusion regimen remain inconclusive.

Liposomal bupivacaine infiltration administered above and below the fascial layer and within the subcutaneous tissue in patients who received intrathecal morphine showed that wound infiltration with liposomal bupivacaine can reduce postoperative pain scores without increasing side effects [57]. However, the opposite result was also reported [58].

In conclusion, local anesthetic wound infiltration and wound infusion have opioid-sparing effects in woman undergoing cesarean delivery under general anesthesia or where intrathecal morphine has been omitted. Subfascial continuous wound infusion is preferable to a single infiltration. Data in women receiving multimodal analgesia, including intrathecal morphine, are sparse and suggest limited benefit [49, 59]. The use of liposomal bupivacaine infiltration requires further evaluation in cesarean delivery.

### 3.6. Bilateral Transversus Abdominis Plane (TAP) Blocks

The TAP block is an abdominal field block between the internal oblique and transversus abdominis muscles that contain 7^th^–11^th^ intercostal nerves and the ilioinguinal and iliohypogastric nerves [60]. In 2008, the first trial investigating bilateral TAP blocks for cesarean delivery was performed with the loss of the resistance technique at the triangle of Petit [61]. All patients received a standard spinal anesthesia with intrathecal fentanyl 25 *μ*g, rectal diclofenac 1 mg/kg, and rectal acetaminophen 1 g at the end of surgery. The results revealed that bilateral TAP blocks provided superior analgesia up to 48 h compared with placebo. The point of injection plays a central role in local anesthetic spreading. A posterior approach to the TAP block provides more spread to the paravertebral space and therefore improved analgesic efficacy compared with the lateral approach [62].

Multiple randomized controlled studies, including the posterior or lateral TAP block for multimodal analgesia, indicated that bilateral TAP blocks had analgesic benefits and opioid-sparing effects compared with placebo. However, the TAP block provides mainly somatic pain but not visceral pain relief, and it has a limited analgesic duration of 6–12 h, whereas intrathecal morphine has analgesic effects up to 36 h. Therefore, compared with intrathecal morphine (100–200 mcg), bilateral TAP blocks provide inferior analgesic efficacy, but they have a lower incidence of opioid-related side effects. In addition, the combination of bilateral TAP blocks to intrathecal morphine did not improve analgesic efficacy or decrease opioid consumption in patients [63]. To overcome the short analgesic effect, liposomal bupivacaine was used for bilateral TAP blocks as part of a multimodal analgesic regimen incorporating 150 *μ*g intrathecal morphine, ibuprofen, and acetaminophen. The bilateral TAP blocks with the liposomal bupivacaine group had a significant opioid-sparing effect of 52% in the first 72 h and 49% at 1 week [64].

However, the TAP block may cause local anesthetic systemic toxicity in cesarean delivery [65, 66]. Obstetric patients are susceptible to local anesthetic toxicity as they have increased sensitivity of nerve axons, higher cardiac output, and less protein binding [67]. Therefore, the minimal effective dose of local anesthetic is highly recommended for this population. A meta-analysis showed no difference in analgesic efficacy between high dose (bupivacaine equivalent >50 mg/side) and low dose (bupivacaine equivalent ≤50 mg/side) [68]. However, because the TAP block is a plane block, the volume of anesthesia should be considered, as it may affect the spreading of local anesthetics and analgesic efficacy. Therefore, the minimum local anesthetic volume is recommended to be ≥ 15 mL per side [69, 70].

In summary, bilateral posterior and lateral approach TAP blocks provide a valuable analgesic option in patients who cannot receive intrathecal morphine. A posterior approach bilateral TAP blocks is preferred over a lateral approach because it provides more effective analgesia. A bilateral TAP blocks may also be used as a rescue technique in patients with severe incisional pain after cesarean delivery.

### 3.7. Bilateral Quadratus Lumborum (QL) Blocks

A QL block is a fascial plane block where a local anesthetic is injected adjacent to the quadratus lumborum muscle into the thoracolumbar fascia layer. The dermatomes that are affected by QL block depend on the approach and vary from T6 to L4 [71–73]. The plausible mechanism of action is to block the thoracic nerves and the sympathetic thoracic trunk of the lower thoracic level [71]. In addition, the thoracolumbar fascia has extensive sensory innervation by both A and C fiber nociceptors and causes sympathetic afferent sympathetic blockade [74]. Because the QL block involves a more posterior approach than the TAP block, the local anesthetic is likely to spread into the paravertebral space. Therefore, the QL block potentially provides analgesia for both somatic and visceral pain and theoretically provides improved analgesia compared to the TAP block [75].

In 2015, the first bilateral QL blocks randomized double-blinded study was conducted to compare bilateral lateral approach QL blocks and control groups in patients undergoing cesarean delivery. All patients in both groups did not receive intrathecal morphine. The patients who received bilateral QL blocks had significantly lower pain scores up to 48 h (VAS at rest: 0 (0-1) vs. 0 (0–3), *P* = 0.004) and lower morphine consumption (48 h morphine use: 11 (4.5–18) vs. 20 (13.0–48), *P* = 0.012) than the control group [76]. A meta-analysis by Xu et al. [77] and Tan et al. [78] showed that bilateral QL blocks provided greater analgesia and reduced postoperative opioid consumption in patients who did not receive intrathecal morphine.

When comparing neuraxial morphine with bilateral QL blocks, Pangthipampai et al. showed that patients who received intrathecal morphine (200 mcg) had lower VAS scores at rest (1 (0–2) vs. 3 [1–5], *P* = 0.011) and lower 24 h morphine consumption (5.5 (0–25) vs. 20 (1–46), *P* = 0.006) than patients who received bilateral posterior approach QL blocks (0.25% bupivacaine 25 mL each side) [79]. Several randomized controlled trials have also reported a greater analgesic efficacy of intrathecal or epidural morphine over bilateral QL blocks [80, 81]. However, one study showed inconsistent results [82]. A meta-analysis found insufficient evidence regarding postoperative opioid use or pain scores with the use of bilateral QL blocks compared with intrathecal morphine [77].

In terms of the addition of a bilateral QL blocks as part of multimodal analgesia, Tamura et al. compared the postoperative analgesic effect in patients who received posterior approach bilateral QL blocks with and without intrathecal morphine (100 mcg) [80]. The results revealed that both groups had comparable analgesic outcomes [80]. Similar results were reported by Irwin et al. [83]. A meta-analysis concluded that the inclusion of bilateral QL blocks as part of multimodal analgesia in patients who received intrathecal or epidural morphine does not provide better analgesia either at rest or during movement at 24 h or lower 24 h morphine consumption [77, 78].

Regarding potential side effects, the peak concentration of local anesthetic is lower after bilateral QL blocks than after bilateral TAP blocks [84]. However, local anesthetics can cause systemic toxicity or hematoma from bleeding because of the presence of lumbar arteries, which are located at the posterior and lateral aspect of the QL muscle. Moreover, lower limb weakness and hypotension have been reported after the QL block due to the local anesthetic spreading to the lumbar plexus [85] and paravertebral space [86]. Therefore, these adverse effects should be considered in patients who received the QL block.

In summary, based on the current knowledge, bilateral QL blocks provided analgesic benefits in patients who did not receive neuraxial morphine. Bilateral QL blocks were shown to reduce opioid consumption and pain scores when compared with bilateral TAP blocks. The addition of bilateral QL blocks to patients who received neuraxial morphine did not improve the analgesic benefits.

The possible advantages and disadvantages of each regional anesthetic technique are given in Table 2.

### 3.8. Nonsteroidal Anti-Inflammatory Drugs (NSAIDs)

NSAIDs are analgesic, antipyretic, and anti-inflammatory drugs that inhibit the cyclooxygenase enzyme (COX) pathway of prostaglandin production. NSAIDs reduce postoperative morphine consumption by 30%–50% after major surgery [89] and cesarean delivery [90, 91], thereby reducing the incidence of opioid-related side effects after surgery. NSAIDs also have very low breast milk transfer, and most NSAIDs are listed by the American Academy of Pediatrics as safe to use during breastfeeding. Therefore, NSAIDs are endorsed by enhanced recovery after surgery (ERAS), the Society for Obstetric Anesthesia and Perinatology (SOAP), and the American College of Obstetrician and Gynecologists (ACOG) for use as part of a multimodal analgesic regimen [15, 16, 92].

Ketorolac is one of the popular intravenous NSAIDs that can be administered via the intravenous or intramuscular (IM) route. In a randomized double-blinded control study of 44 elective cesarean deliveries, intravenous ketorolac 30 mg reduced the 24 h use of morphine by 31.7% [93]. Parecoxib is another intravenous NSAID that has been approved in European and Asian countries and in Mexico. A single dose of intravenous parecoxib did not reduce postoperative morphine consumption, but it reduced postoperative pain scores with higher patient satisfaction [94]. With respect to NSAIDs in oral or suppository form, naproxen [95], ibuprofen [96], celecoxib [97], and diclofenac suppositories [98, 99] are mostly effective compared with placebo (Table 3). However, there are no studies that compare the analgesic efficacy of different NSAIDs.

In summary, for women undergoing cesarean delivery, scheduled NSAIDs should be administered in the postpartum period in the absence of contraindications. The type of NSAIDs should be based on the patient's condition (e.g., a history of dyspepsia), drug availability, and drug safety profile while breastfeeding [15, 16, 92, 100].

### 3.9. Acetaminophen

Acetaminophen is the most common analgesic used worldwide and has a long record of safe use and few side effects. Acetaminophen inhibits peroxidase, leading to a reduction in prostaglandin formation [108]. Therefore, acetaminophen has analgesic and antipyretic effects. The mechanism of action of acetaminophen is also proposed as interference with the descending serotonergic pain pathways and weak binding to cannabinoid receptors, which inhibits nitric oxide production in the spinal cord and modulates nociceptive transmission [109].

The inclusion of acetaminophen in multimodal analgesia produces opioid-sparing effects. A significant reduction in 24 h morphine consumption is observed with acetaminophen compared with placebo after major surgery [89] and cesarean delivery [110]. A summary of the relevant studies is given in Table 4. Therefore, acetaminophen has been recommended as a component of postcesarean delivery analgesia in various guidelines [15, 16, 92, 100] due to its safety profile at regular doses [111]; improved efficacy of analgesia, especially when it is combined with NSAIDs [112, 113]; and reduced breast milk penetration [114].

In a retrospective study of patients who received intrathecal morphine and scheduled acetaminophen for 48 h, patients who received scheduled oral acetaminophen needed less intravenous morphine than the as-needed group (13.8 ± 14.3 vs. 23.0 ± 17.7 mg, *P* ≤ 0.001) [115]. Comparing oral and intravenous acetaminophen, a randomized controlled trial of 141 patients undergoing cesarean delivery showed no difference in opioid consumption between groups but reduced opioid consumption when compared with those who received no acetaminophen [116].

Because combining acetaminophen and NSAIDs has an additive analgesic effect, both drugs should be administered routinely after cesarean delivery [15, 16, 92, 100, 113]. Intravenous forms of both acetaminophen and NSAIDs are not recommended, as they lack clear evidence and cause higher costs. Intravenous administration should be reserved for patients who cannot tolerate oral intake or those who develop nausea or vomiting.

### 3.10. Steroids

Steroids are well known as the drug of choice for the prevention of postoperative nausea and vomiting [121]. Moreover, steroids also have an analgesic property by inhibiting the conversion of phospholipase A2 to arachidonic acid, which is the precursor of prostaglandin formation.

Four randomized controlled trials evaluated the use of intravenous dexamethasone 8–10 mg [122–125]. The results revealed that intravenous dexamethasone reduced modest pain scores, improved patient recovery outcomes [126], and prolonged postoperative analgesia [122] in patients undergoing cesarean section under spinal anesthesia. A meta-analysis of patients who received neuraxial morphine, including four trials of cesarean delivery and four abdominal hysterectomies, showed that a single dose of dexamethasone decreased pain scores compared with the placebo (mean difference (95% CI = −0.30 (−0.46, −0.13)) and reduced the use of rescue analgesics (RR (95% CI) = 0.72 (0.52, 0.98)) [127]. However, the side effects of dexamethasone include elevated postoperative blood glucose levels, increased risk of wound infection, and delayed wound healing. A meta-analysis reported that single dose dexamethasone did not increase the incidence of delayed wound healing or increase the risk of infection [127]; nevertheless, dexamethasone should be avoided in patients with insulin resistance. The effect of elevated blood glucose levels appeared to be increased in a dose-dependent manner.

Thus, eventhough single dose dexamethasone did not clinically improve pain scores, it reduced the need for rescue analgesia by 30% and had antiemetic properties [127]. Therefore, the procedure-specific postoperative pain management (PROSPECT) guidelines recommended using a single intravenous dose of dexamethasone for cesarean delivery in the absence of contraindications [100]. However, other guidelines still do not endorse intravenous dexamethasone in routine use [15, 16, 92]. The risks and benefits should be evaluated in terms of the use of steroids.

### 3.11. Ketamine

In recent years, multiple research trials have suggested the usefulness of ketamine as a strong analgesic when used in subanesthetic intravenous doses. The proposed mechanism of ketamine is the blockade of postsynaptic N-methyl-D-aspartate (NMDA) receptors, neuronal hyperpolarization-activated cationic currents, nicotinic acetyl-choline ion channels, and delta and mu-opioid receptors [128]. Ketamine may also reduce cholinergic neuromodulation [129, 130] and enhance the inhibitory serotoninergic pathway [131].

In 2005, the first subanesthetic intravenous doses of ketamine (0.15 mg/kg) were administered to patients undergoing cesarean delivery under spinal anesthesia. The results revealed that ketamine prolonged the time to the first analgesic requirement (53 min) and decreased the total analgesic consumption and pain score [132]. Han et al. used a larger dose of ketamine (0.5 mg/kg intravenous bolus, followed by 0.25 mg/kg/h continuous infusion) during surgery [133]. In the ketamine group, there was significantly less fentanyl use at 2 h after surgery (58.0 ± 27.5 vs. 81.2 ± 30.4 mg, *P* = 0.033) but no statistically significant difference at 6, 24, or 48 h after surgery. Pain scores at 2, 6, 24, and 48 h were comparable between groups [133]. Bauchat et al. conducted a randomized controlled trial of ketamine 10 mg IV as part of a multimodal analgesia regimen (intrathecal morphine 150 *μ*g and ketorolac 30 mg IV every 6 h) [134]. The pain score and 24 h opioid consumption were not different at 24, 48, or 72 h. However, at 2 weeks postpartum, the ketamine group had lower pain scores than the control group (difference −0.6, 95% CI −1.1 to −0.9). Regarding side effects, more patients in the ketamine group reported being drowsy, restless, lightheaded, dizzy, or having double vision [134].

A recent meta-analysis evaluated the analgesic effect of low doses of ketamine in 20 cesarean delivery studies (general anesthesia was administered in seven studies and spinal anesthesia in five studies) [135]. The results revealed that ketamine enhanced postoperative analgesia for 49.36 min (95% CI 43.31–55.41) after cesarean delivery under spinal anesthesia. Visual analogue scale pain scores at rest 2 h after surgery were significantly lower in the ketamine group, and no differences were noted in maternal nausea, vomiting, pruritus, or psychommetric effects between groups [135].

Currently, ketamine is not recommended as a routine drug for postoperative pain strategies. However, the addition of ketamine as part of a multimodal regimen may be effective in patients with escalating opioid requirements or in women with a history of chronic pain [136].

### 3.12. Gabapentinoids

Gabapentinoids inhibit the *α*_2_*δ* subunit of calcium channels and enhance the inhibitory serotoninergic pathway. Gabapentinoids are the most commonly used to manage chronic neuropathic pain. Their use in the perioperative period was proposed, as trials suggested that gabapentinoids may have a protective effect and prevent persistent postsurgical pain [137] and reduce opioid consumption in the early postoperative period [138].

Gabapentinoids as an adjunct analgesic for cesarean delivery have been evaluated. In a randomized control study, preoperative oral gabapentin 600 mg was administered 1 h before surgery as part of a multimodal analgesia regimen (intrathecal morphine 100 mcg, oral diclofenac 50 mg every 8 h, and acetaminophen 1 g every 6 h). The pain score (visual analogue scale 0–100 mm) on movement at 24 h was 21 mm (95% CI 13–28) in the gabapentin group and 41 mm (95% CI 31–50) in the placebo group (*P* = 0.001), without a significant difference in opioid consumption. Severe maternal sedation was observed more often in the gabapentin group (19% vs. 0%, *P* = 0.04) [139]. Monks et al. used a larger dose of oral gabapentin 600 mg preoperatively followed by 200 mg every 8 h for 2 days [140]. The results revealed that there was a small reduction in pain score (−7 mm (−13 to 0); *P* = 0.047) with greater patient satisfaction in the gabapentin group (87 vs. 77 mm, *P* = 0.003) [140]. However, gabapentin produced a significantly higher incidence of sedation (55% vs. 39%, *P* = 0.026) [140]. In contrast, Short et al. reported no significant analgesic benefits with gabapentin compared with placebo [141]. A meta-analysis of cesarean delivery under spinal anesthesia reported that gabapentin significantly reduced the pain score on movement at 24 h (mean difference −11.58, 95% CI −23.04 to −0.12). However, pain scores at other time points at rest or on movement were not significantly different [142].

There are several limitations of gabapentinoid use. First, gabapentinoids have a high umbilical vein-to-maternal vein ratio. Therefore, gabapentinoids should be avoided as preemptive administrations in patients undergoing cesarean delivery [139]. Second, gabapentinoids cause maternal side effects (e.g., sedation and visual disturbance). Moreover, current evidence still fails to demonstrate a strong benefit of gabapentinoids on postoperative pain in cesarean delivery, as well as the potential adverse effects and neonatal safety profile [143]. Therefore, gabapentinoids are still not recommended for routine use in postcesarean analgesia. However, they can be considered as a part of multimodal analgesia to decrease opioid consumption or improve pain relief in patients with chronic pain [136].

### 3.13. Evaluation of Recovery Function beyond the Pain Score

Effective postoperative pain management is paramount for faster recovery. A good pain score does not imply that the patient has good functional recovery. As an example, a prospective observational study using activity trackers in women who underwent vaginal delivery and women who underwent cesarean delivery revealed similar pain scores. Vaginal delivery was associated with greater early ambulation than cesarean delivery. This observation confounds the importance of using pain scores or opioid consumption as the prime quality of care indicators in obstetric anesthesia and analgesia.

Enhanced functional recovery is becoming a prime success indicator of modern perioperative healthcare [144]. The Quality-of-Recovery (QoR-40) score [145] and QoR-15 [146] have been extensively studied to measure the recovery outcome following general surgery. However, neither tool is focused on the obstetric population. To date, the global measure of patient outcomes focusing on obstetric patients, namely, the “Obstetric Quality-of-Recovery (ObsQoR-11) score ,” includes evaluations of physical comfort, pain relief, physical independence, emotional state, and ability to care for the baby [17, 19]. However, ObsQoR-11 has been updated to ObsQoR-10 by combining severe and moderate pain items, based on the patient feedback. ObsQoR-10 has been validated following spontaneous, instrumental, vaginal, and cesarean delivery in multiple healthcare setting [18, 147, 148]. However, more studies are needed to validate translated versions and determine minimal important clinical change and clinically significant differences in scores.

Postpartum pain and functional recovery were more comprehensively assessed in obstetric patients. Komatsu et al. conducted a prospective observational study of 213 nulliparous patients after vaginal or cesarean delivery [5]. The patients were assessed daily until they achieved three outcomes: [1] pain resolution, [2] opioid cessation, and [3] self-assessed functional recovery from delivery. In women who underwent cesarean section, the median times to pain resolution and to functional recovery to the prepartum levels were longer than those in women who underwent vaginal delivery (time to pain resolution: 21 (IQR 14–27) vs. 14 (IQR 7–24) days; time to functional recovery: 27 (IQR 19–40) vs. 19 (IQR 11–24) days) [5]. Pain was strongly correlated with the time of functional recovery, which was 1.7 times greater in women who underwent cesarean delivery. This provided more detail regarding recovery to predelivery levels of functioning, which appears to occur mainly by pain resolution, and opioid use is more apparent after cesarean delivery than after vaginal delivery.

## 4. In Conclusion

Stepwise multimodal analgesia has been confirmed to be effective in pain management and opioid-sparing effects. The regimens currently recommended by the ERAS, SOAP, and PROSPECT guidelines are given in Table 5. Optimal intraoperative and postoperative neuraxial analgesia has always been the focus for patient undergoing cesarean delivery. Significant pain is associated with delayed recovery, poor clinical outcomes, and poor maternal–fetal bonding. The prescribed postoperative analgesic regimen should be individualized based on preoperative risk stratification for severe pain and analgesic-related adverse effects—for example, a patient with chronic pain undergoing cesarean delivery under neuraxial anesthesia. Postoperative epidural analgesia, QL blocks, or adjunct medications (e.g., ketamine or gabapentinoids) may be beneficial to optimize analgesia and clinical outcomes.

**Figure 1:**
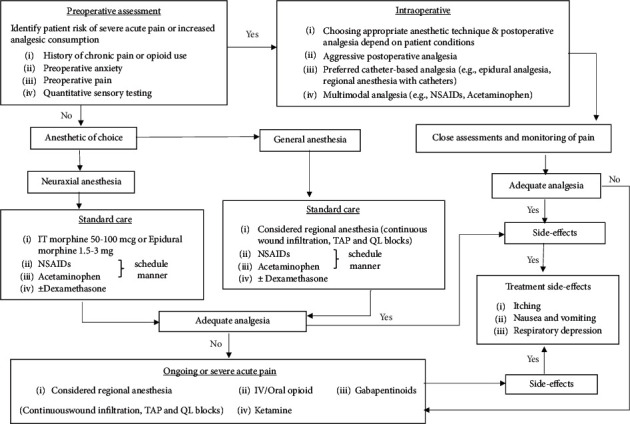
Stepwise multimodal analgesia for postcesarean delivery pain control.

Stepwise multimodal protocols are recommended to reduce postoperative opioid consumption. The general approach and analgesic recommendation in patients undergoing cesarean delivery with neuraxial anesthesia include intrathecal morphine in conjunction with scheduled NSAIDs and acetaminophen (Figure 1). Additional opioid administration is reserved for breakthrough pain to avoid the risk of drug transfer to breastfeeding neonates. Further investigation is required to determine analgesic drugs or dose alterations based on preoperative predictions for patients at risk of severe pain. Outcomes beyond pain and analgesic use, such as functional recovery, should be determined to evaluate analgesic treatment regimens.

## Figures and Tables

**Table 1:** Risk factors for severe postoperative pain after cesarean delivery.

Patient related factors	Questionnaire and quantitative sensory testing	Perioperative risk factors
Preoperative anxiety [28, 29] Previous cesarean delivery [30] History of chronic pain or history of chronic opioid use [20]	Quantitative sensory testing: electrical pain threshold [31], heat pain threshold [32], and local infiltration [21]Three simple questions (level of anxiety, anticipated pain, and analgesics need) [25]	Elevated numerical rating score for pain at first 24 hours [33]

**Table 2:** Comparison efficacy of regional anesthetic techniques on analgesic efficacy.

	Possible advantages	Possible disadvantages
Single-shot local anesthetic wound infiltration	Easy to perform May benefit patients who did not receive intrathecal morphine [49]	Provides only somatic pain relief Limited duration of action: 4–12 h [51, 52]

Continuous wound infusion	Decreased opioid consumption [53]	Provides only somatic pain relief Risk of leakage [53] Risk of dislodge [53]

Bilateral transversus abdominis plane blocks	Decreased opioid consumption [63] Duration of action: 6–12 h [63]	Provides only somatic pain relief [60] Higher risk of local anesthetic systemic toxicity than with other techniques [65, 84] Risk of block-related side effects

Bilateral quadratus lumborum blocks	Decreased opioid consumption [77, 78] Duration of action up to 24–48 h [76, 87]	Provides somatic ± visceral pain relief [88] Risk of block-related side effects

**Table 3:** Randomized controlled studies evaluating efficacy of nonsteroidal anti-inflammatory drugs (NSAIDs) for postcesarean delivery analgesia.

Author (year)	Sample size	Intrathecal opioid	Analgesic regimen	Comparison groups	Pain score	Opioid consumption	Opioid-related side effects	Conclusion
Cohen (1996) [35]	48 elective cesarean deliveries under spinal anesthesia	MO 0.1-0.2 mg	Ketorolac IV 60 and then 30 q 6 h × 3 doses	Four groups (i) Group 1: spinal morphine 0.1 mg (ii) Group 2: spinal morphine 0.2 mg (iii) Group 3: spinal morphine 0.1 mg + ketorolac (iv) Group 4: ketorolac alone	No difference in pain score among the groups	20 h meperidine consumption (i) Group 1: 72 ± 22 mg (ii) Group 2: 46 ± 21 mg (iii) Group 3: 39 ± 11 mg (iv) Group 4: 49 ± 15 mg, no statistical differences	Less pruritus in group 4	Ketorolac provides satisfactory analgesia with few side effects

Pavy (2001) [91]	44 elective cesarean deliveries under CSE	Fent 12.5 *μ*g	Ketorolac IV 30 mg at PACU and then 120 mg drip in 24 h. In postoperative day 1, initial ketorolac 15 mg IV bolus and then 105 mg IV drip in 24 h	Two groups (i) Group 1: ketorolac group (ii) Group 2: placebo	(i) No difference in pain with movement at 12, 24 48, or 72 h. (ii) Worst pain score (VAS) at 12 h, group 1 : 38 [20, 50] and group 2 : 60 [43, 73], *P* 0.003	First 12–24 h meperidine use in mg (median (IQR)) (i) Group 1: 105 (57, 150) (ii) Group 2: 150 (108, 226), *P* 0.012	The severity of pruritus, sedation, and nausea did not differ between groups.	Intravenous ketorolac produced a meperidine dose-sparing effect approximately 30% but did not significantly improve pain relief, reduce opioid-related side effects, or change patient outcomes

El-Tahan (2007) [101]	90 elective cesarean deliveries under GA	No IT opioid	Ketorolac IV 15 mg bolus 20 min before induction and then drip 7.5 mg/h	Two groups (i) Group 1: ketorolac group (ii) Group 2: placebo	VAS score (i) Group 1: at rest, 2 (0–6); on movement, 5 [3–9] (ii) Group 2: at rest, 4 [3–7]; on movement, 7 [6–10], *P* ≤ 0.001	Number receiving tramadol first 4 h: (i) Group1: 31.1% (ii) Group2: 15.6%, *P* 0.004	The frequency and severity of sedation or N/V did not differ between groups.	Prophylactic ketorolac is safe and improves the quality of analgesia after cesarean delivery.

Khezri (2018) [102]	150 elective cesarean deliveries under spinal anesthesia	No IT opioid	Ketorolac IV 30 mg (10 min before spinal anesthesia)	Three groups (i) Group 1: ketorolac group (ii) Group 2: meperidine group (iii) Group 3: placebo	Mean time to first analgesia request was significantly longer in groups 1 and 2 compared with group 3.	The 24 h analgesic consumption in groups 1 and 2 was significantly smaller than group 3 (*P* < 0.001). However, there were no significant differences between group 1 and 2 (*P* 0.41).	—	Preemptive IV meperidine and ketorolac can provide a satisfying analgesia immediately after surgery.

Lowder (2003) [93]	44 cesarean delivery patients	N/A	Ketorolac 30 mg IV postoperative period	Two groups (i) Group 1: ketorolac group (ii) Group 2: placebo group	Pain scores were significant different at 2,3,4,6,12, or 24 h, *P* 0.033	24 h MO equivalents consumption was lower in the ketorolac group. (i) Group 1: 28.1 ± 3.35 mg (ii) Group 2: 41.6 ± 4.25 mg, *P* 0.008	—	Ketorolac is efficacious in reducing postoperative pain and narcotic usage after cesarean delivery.

Alhashemi (2006) [96]	45 elective cesarean deliveries under spinal anesthesia	Fent 10 *μ*g	Ibuprofen 400 mg oral *q* 6 h for 48 h; first dose 30 min before surgery	Two groups (i) Group 1: acetaminophen 1 gm IV *q* 6 *h* + ibuprofen (ii) Group 2: acetaminophen 1 g IV *q* 6 *h* + placebo	No difference of VAS between groups, *P* 0.143.	No difference in MO requirement (i) Group 1: 93 ± 33 mg (ii) Group 2: 98 ± 37 mg, *P* 0.628	Incidence of pruritus was higher in group 1 (45.5% vs. 82.6%, p 0.031). No difference in the incidence of N/V.	IV acetaminophen is an alternative to oral ibuprofen as an adjunct to MO PCA after cesarean delivery.

Angle (2002) [95]	80 elective cesarean deliveries under spinal anesthesia	MO 0.2 mg + Fent 10–20 *μ*g	Naproxen 500 mg supposition then oral 550 mg *q* 12 h × 6 doses. Every patient received acetaminophen 300 mg + caffeine 15 mg + codeine 30 mg, 1-2 tab, PRN 3-4 h.	Two groups (i) Group 1: naproxen group (ii) Group 2: placebo group	Incisional pain on sitting at 36 h. (i) Group 1: 38.2 ± 26 (ii) Group 2: 51.4 ± 25.7, *P* 0.05	Opioid use over time significantly less in the naproxen group, *P* < 0.01	No difference in the incidence of pruritus, N/V, maternal sedation, or respiratory rates	Adding regular doses of naproxen to spinal MO leads to improved analgesia on postoperative day 1.

Sun (1992) [103]	120 elective cesarean deliveries under epidural anesthesia	Epidural MO 2 mg	Diclofenac 75 mg IM on arrival in the recovery room	Four groups (i) Group 1: diclofenac IM + epidural saline (ii) Group 2: epidural MO 2 mg + NSS (iii) Group 3: epidural MO 2 mg + diclofenac IM (iv) Group 4: epidural and IM saline	Overall pain relief was better in group 3 compared with other groups (*P* < 0.05).	Total meperidine consumption (i) Group 1: 2450 mg (ii) Group 2: 400 mg (iii) Group 3: 0 (iv) Group 4: 3650 mg	Incidence of N/V and pruritus occur more frequently in groups 2 and 3 (*P* < 0.05).	Combined epidural MO 2 mg and diclofenac IM enhances analgesic efficacy in the treatment of both wound pain and uterine cramps

Bush (1992) [104]	50 elective cesarean deliveries under GA	—	Diclofenac IM 75 mg single dose before discontinuing anesthesia	Two groups (i) Group1: diclofenac group (ii) Group2: placebo group	Linear analogue scores (LAS) for pain were significantly lower in group 1 at 6 h after surgery. (i) Group 1: 0.5 (0.2–2.0) (ii) Group 2: 2.0 (0.1–3.5), *P* < 0.05. However, no difference in LAS at 12 h	Cumulative papaveretum consumption at 18 h was lower in patient who received diclofenac. (i) Group 1: 61.4 ± 30.2 mg (ii) Group 2: 91.4 ± 23.4 mg, *P* < 0.05	No difference in the incidence of sedation scores or N/V by 12 h	Giving diclofenac enhances their effectiveness as analgesics.

Olofsson (1999) [99]	50 elective cesarean deliveries under spinal anesthesia	No IT opioids	Diclofenac 50 mg rectal × 3 doses in 24 h	Two groups (i) Group 1: diclofenac group (ii) Group 2: placebo group	VAS score during first 3 h postoperative was lower in group 1 than group 2, *P* 0.025	Total delivered doses of ketobemidone (i) Group 1: 30.9 ± 3.3 mg (ii) Group 2: 47.6 ± 3.08 mg, *P* < 0.01	N/A	Adding diclofenac during first 24 h reduces the need for opioids with the improved analgesic effect.

Dahl (2002) [98]	82 elective cesarean deliveries under spinal anesthesia	N/A	Diclofenac 100 mg rectal *q* 12 h	Two groups (i) Group 1: diclofenac group (ii) Group 2: placebo group	No difference in VAS	Accumulative 32 h MO consumption was less in the diclofenac group (i) Group 1: 14 ± 1.5 mg (ii) Group 2: 21.5 ± 1.6 mg, *P* < 0.05	Incidence of N/V during first 8 h was higher in the placebo group. (i) Group 1: 0% (ii) Group 2: 11.9%	Diclofenac suppositories 100 mg given twice daily after cesarean section are opioids sparing.

Wilder-Smith (2003) [105]	120 elective cesarean deliveries under spinal anesthesia	No IT opioid	Diclofenac 75 mg IM	Four groups (i) Group 1: diclofenac 75 mg IM (ii) Group 2: tramadol 100 mg IM (iii) Group 3: diclofenac 75 mg + tramadol 100 mg IM (iv) Group 4: placebo	Lower pain intensity ratings at rest when comparing group 3 with group 1 (at 30 min, 6 h, and 7 h postinjection; *P* < 0.04) and group 4 (at 30 and 60 min and 6 and 7 h; *P* < 0.05).	The total rescue morphine (i) Group 1: 31 (95% CI 26–36) mg (ii) Group 2: 35 (95% CI 32–38) mg (iii) Group 3: 28 (95% CI 24–33) mg (iv) Group 4: 38 (95% CI 35–41) mg, *P* < 0.005	No difference in the incidence of N/V or sedation score in all groups	The combination of tramadol and diclofenac resulted in improved analgesia compared with monotherapy

Bourlert (2005) [106]	64 cesarean deliveries	N/A	Diclofenac 75 mg IM single dose	Two groups (i) Group 1: diclofenac group (ii) Group 2: placebo group	No difference of VAS score at 1, 2, 6, and 20 h	Mean use of MO was less in the diclofenac group Group 1: 21.69 ± 9.78 mg Group 2: 27.4 ± 11.09 mg, *P* 0.016	N/A	A single dose of diclofenac IM decreases the use of morphine during the postcesarean delivery

Thienthong (2012) [107]	30 elective cesarean deliveries	MO 0.2 mg	Diclofenac 75 mg IV drip at 12 h after surgery	2 groups (i) Group 1: diclofenac group (ii) Group 2: placebo group	24 h mean pain score was not statistical difference	No difference in postoperative tramadol consumption between groups	No difference in incidence of N/V or abdominal discomfort	Intramuscular diclofenac 75 mg for IV route in combination with spinal MO 0.2 mg provides good analgesia within 24 h after cesarean delivery

Matsota (2013) [97]	64 elective cesarean deliveries under CSE	Fent 200 *μ*g	Celecoxib 200 mg oral OD every patient received PCEA 0.15% ropivacaine + fent 2 mcg/ml bolus 4 ml with lockout period 15 min	2 groups (i) Group 1: celecoxib group (ii) Group 2: control group	The VAS scores at rest and movement were constantly lower in group 1.	No difference in the attempted doses or the total volume of the local anesthetic administration between two groups.	No difference in incidence of dizziness, sleepiness, bladder dysfunction, itching, or vomiting between the 2 groups.	A single dose of 200 mg of celecoxib effectively improved pain management in parturient with PCEA.

Inthigood (2017) [94]	82 elective cesarean deliveries under spinal anesthesia	MO 0.2 mg	Parecoxib 40 mg IV single dose	Two groups (i) Group 1: parecoxib group (ii) Group 2: placebo group	The VAS scores at rest were lower in the parecoxib group. Median VAS (IQR) (i) Group 1: 1 (0, 2) (ii) Group 2: 2 (0.5, 3), *P* 0.01	Total meperidine consumption was no statistically difference. (i) Group 1: 8.3 ± 16.7 (ii) Group 2: 12.7 ± 18.8, *P* 0.27	No patients in either group reported adverse effects from their assigned intervention	Parecoxib did not demonstrate effectiveness in reducing patient requirement for supplementary meperidine after cesarean delivery. However, administration of a single 40 mg dose of IV parecoxib after elective cesarean delivery demonstrated effectiveness in reducing pain scores

^*∗*^All analgesics are administered postoperatively unless indicated. All visual analogue scale or postoperative morphine consumption are reported as mean ± standard deviation (SD) unless otherwise specified. CSE, combined spinal epidural anesthesia; Fent, fentanyl; GA, general anesthesia; h, hour; IM, intramuscular; IQR, interquartile range; IT, intrathecal; IV, intravenous; min, minute; MO, morphine; N/A, not applicable; N/V, nausea and vomiting; PACU, postanesthesia care unit; PCA, patient-controlled analgesia; PCEA, patient-controlled epidural analgesia; VAS, visual analogue scale.

**Table 4:** Randomized controlled studies evaluating administration of acetaminophen for postcesarean delivery analgesia.

Author (year)	Sample size	Intrathecal opioid	Analgesic regimen	Comparison groups	Pain score	Opioid consumption	Opioid-related side effects	Conclusion
Siddik (2001) [117]	80 elective cesarean deliveries under spinal anesthesia	Fent 12.5 *μ*g	Acetaminophen 2 g IV q 6 h Diclofenac 100 mg rectal q 8 h	Four groups (i) Group 1: acetaminophen group (ii) Group 2: diclofenac 100 mg supposition q 8 h (iii) Group 3: propacetamol + diclofenac (iv) Group 4: placebo group	At 2 h, VAS both at rest and on cough were significantly lower in groups 2 and 3 compared with group 4.	MO consumption at 2,6, and 24 h was significantly lower in groups 2 and 3 than in groups 1 and 4. 24 h MO consumption; (i) Group 1: 61.1 ± 23 mg (ii) Group 2: 36 ± 18 mg (iii) Group 3: 28.3 ± 15.8 mg (iv) Group 4: 66.7 ± 20 mg	Incidence of nausea, vomiting, excessive sedation, and pruritus was similar for all groups. No patient had respiratory depression.	Adding acetaminophen to diclofenac improves analgesia and has a highly significant morphine sparing effect.

Paech (2013) [118]	111 elective cesarean deliveries under CSE	Fent 15 *μ*g	Acetaminophen IV 2 g and then 1 g oral *q* 6 h	Four groups (i) Group 1: acetaminophen 2 g IV then 1 gm oral *q* 6 h *×* 3 doses (ii) Group 2: Parecoxib 40 mg IV then celecoxib 400 mg at 12 h (iii) Group 3: Control group	No difference in 24 and 48 h pain at rest or with movement	No difference in total postoperative meperidine consumption	No difference in incidence of N/V or severity of sedation between groups incidence, and severity of pruritus was greater in the treatment groups than in the control group.	Addition of regular acetaminophen, COX-2 inhibitors, or both to meperidine. PCEA did not exhibit a meperidine dose-sparing effect during the first 24 h

Ozmete (2016) [119]	60 elective cesarean deliveries under GA	—	Acetaminophen 1 g IV before induction of anesthesia + IV PCA morphine	Two groups (i) Group 1: acetaminophen group (ii) Group 2: placebo group	Median VAS scores were significantly lower in group 1 than in group 2 at all postoperative time points except for the score at 24 h postoperatively	24 h MO consumption (i) Group 1: 24 (IQR: 14–31) mg (ii) Group 2: 38 (IQR: 26–46) mg, *P* ≤ 0.001	No difference in the incidence of sedation or nausea	Preoperative acetaminophen 1 g IV single dose effectively decreased in pain reduction and opioid requirement within 24 h after cesarean delivery

Alteau (2017) [110]	104 cesarean deliveries under regional anesthesia	N/A	Acetaminophen 1 g IV q 8 h. First dose begins 30–60 min before skin incision.	Two groups (i) Group 1: acetaminophen group (ii) Group 2: placebo group	No difference in pain score	24 h MO consumption was lower in the acetaminophen group (47 ± 39.1 mg) than in the placebo group (65 ± 46.24 mg), *P* < 0.034	No difference in incidence of N/V, respiratory depression, or constipation	IV acetaminophen reduces oral narcotic consumption after cesarean delivery

Tower (2018) [120]	105 elective cesarean deliveries under spinal anesthesia	Fent 20 *μ*g + MO 0.2 mg	Acetaminophen 1 g IV prior to spinal block	Two groups (i) Group 1: acetaminophen group (ii) Group 2: placebo group	No difference in pain score	No difference in opioid requirement (i) Group 1: 94.2 ± 40.4 mg (ii) Group 2: 90.7 ± 42.1 mg, *P* 0.67	N/A	Preoperative IV acetaminophen single dose did not reduce pain score or postoperative opioid consumption

^*∗*^All analgesics are administered postoperatively unless indicated. All visual analogue scale or postoperative morphine consumption are reported as mean ± standard deviation (SD) unless otherwise specified. COX, cyclooxygenase enzyme; CSE, combined spinal epidural anesthesia; Fent, fentanyl; GA, general anesthesia; g, gram; h, hour; IQR, interquartile range; IV, intravenous; mg, milligram; MO, morphine; N/A, not applicable; PCA, patient-controlled analgesia; VAS, visual analogue scale.

**Table 5:** Postoperative analgesic recommendation for cesarean delivery.

	SOAP consensus statement [6]	ERAS society [7]	PROSPECT guideline [37]
Morphine	Neuraxial long-acting opioid example: Intrathecal morphine 50–150 mcg or epidural morphine 1–3 mg	Long-acting intrathecal opioids such as morphine provides analgesia for several hours after cesarean delivery, although the expense of a number of side effects include nausea, vomiting, and pruritus.	Intrathecal morphine 50–100 mcg or diamorphine 300 mcg. Epidural morphine 2-3 mg or diamorphine 2-3 mg may be administered as an alternative.

Acetaminophen and NSAIDs	NSAIDs analgesia started in OR unless contraindicated: (i) Ketorolac 15–30 mg IV after peritoneum closed (ii) Acetaminophen IV after delivery or orally, per oral before or after delivery	Regular NSAID and acetaminophen are recommended for enhanced recovery for cesarean delivery.	Prescribe acetaminophen and a NSAID administered after delivery and continued regularly postoperatively.

Dexamethasone	—	—	A single dose of IV dexamethasone after delivery in the absence of contraindication

Local anesthetic techniques	Consider local anesthetic wound infiltration or regional blocks such as bilateral TAP or QL blocks if neuraxial morphine is not administered.	In the absence of long-acting intrathecal opioids, the TAP field block provides excellent postoperative pain control. A Cochrane review of local analgesia infiltration and abdominal nerve blocks found that they improved postoperative analgesia for cesarean delivery.	Consider a single injection of local anesthetic infiltration, continuous wound local anesthetic infusion, and/or fascial plane blocks, if intrathecal morphine is not administered.

ERAS, enhanced recovery after surgery; IV, intravenous; NSAID, nonsteroidal anti-inflammatory drug; PROSPECT, procedure-specific postoperative pain management; OR, operating room; QLB, quadratus lumborum block; SOAP, Society for Obstetric Anesthesia and Perinatology; TAP, transversus abdominis plane block.

## Data Availability

The reference data supporting this review article are from previously reported studies and datasets, which have been cited.
